# Fragility analysis and systematic review of patellar resurfacing versus non‐patellar resurfacing in total knee arthroplasty

**DOI:** 10.1002/jeo2.12113

**Published:** 2024-08-06

**Authors:** Teja Polisetty, Alexandra L. Hohmann, Eric DiDomenico, Jess H. Lonner

**Affiliations:** ^1^ Department of Orthopaedics Harvard University Cambridge Massachusetts USA; ^2^ Rothman Orthopaedic Institute at Thomas Jefferson University Philadelphia Pennsylvania USA

**Keywords:** arthroplasty, fragility, joint arthroplasty, randomised controlled trials, knee, patellar resurfacing

## Abstract

**Introduction:**

Fragility analysis is a method of further characterising the robustness of statistical outcomes. This study evaluates the statistical fragility of randomised controlled trials (RCTs) comparing patellar resurfacing versus non‐patellar surfacing in total knee arthroplasty (TKA).

**Methods:**

PubMed, MEDLINE and EMBASE were searched for RCTs comparing outcomes in TKA based on patellar resurfacing. Fragility index (FI) and reverse FI (collectively, “FI”) were calculated for dichotomous outcomes as the number of outcome reversals needed to change statistical significance. Fragility quotient (FQ) was calculated by dividing the FI by the sample size for that outcome. Median FI and FQ were calculated for each individual outcome and for the overall study. Subanalyses were performed to assess FI and FQ based on outcome type, statistical significance and loss to follow‐up.

**Results:**

Twenty‐one RCTs were included in the analysis, capturing 3910 subjects. The overall median FI was 5.0 (interquartile range, [IQR] 4.0−6.0), and the overall median FQ was 0.048 (IQR 0.022−0.065). The outcome of anterior knee pain has a median FI of 6.0 (IQR 4.0−6.0) and a median FQ of 0.057 (IQR 0.025−0.065). Only five (7%) outcomes were significant. The loss to follow‐up was greater than the FI in 12 of 19 studies (63%) with available data.

**Conclusion:**

RCTs comparing patellar resurfacing in TKAs show significant statistical fragility; a few outcome reversals can alter findings. The majority of outcomes were nonsignificant, indicating that the choice to resurface the patella may not affect most clinical outcomes; however, clinical conclusions are limited by the statistical fragility of the analysed outcomes. Larger RCTs for this comparison are necessary, and we suggest adding FI and FQ to RCT reports with *p* values to improve the interpretability of results.

**Level of Evidence:**

Level II.

**Abbreviations**
FI: fragility index
FQ: fragility quotient
IQR: interquartile range
QUALY: quality‐adjusted life years
RCT: randomised controlled trial
RFI: reverse fragility index
TKA: total knee arthroplasty

## INTRODUCTION

Total knee arthroplasty (TKA) is a standard procedure for advanced knee osteoarthritis. However, there remains uncertainty among orthopaedic surgeons about the effectiveness, necessity and risk of resurfacing the patella during this surgery [8, 52]. Randomised controlled trials (RCTs) comparing TKA with or without patellar resurfacing have shaped practice philosophies regarding patellar resurfacing despite varied conclusions [4, 6, 38, 51, 52]. A meta‐analysis of 13 RCTs found rates of anterior knee pain rate to be the same at 10% in TKA with or without resurfacing [37]. Nonetheless, many surgeons endorse routine or selective patellar resurfacing and cite studies showing higher reoperation rates ranging from 1.4% to 8% in TKA performed without patellar resurfacing [35, 36, 39]. Concerns about the possibility of patellar fractures and other complications related to a resurfaced patella have prompted some surgeons to favour not resurfacing the patella [38, 43]. Secondary patellar resurfacing has also shown unpredictable and often poor results, with over 50% of patients still experiencing anterior knee pain postprocedure and 65% of patients not satisfied [47]. Further analysis of existing research into patellar resurfacing in TKA may clarify areas of uncertainty.

The use of the fragility index (FI), an emerging statistical analysis technique, may help facilitate this analysis. First applied to clinical trials by Walsh et al., the FI is a measure of the minimum number of reversals of events to non‐events to convert an outcome from statistically significant to nonsignificant [50]. Inversely, a reverse FI may be calculated to determine the number of event changes necessary to convert an outcome from nonsignificant to significant [25]. Since FI has been demonstrated to be correlated with sample size and event rates, in order to appropriately compare FI values across different trials, the FI may be divided by the study population size to calculate a fragility quotient (FQ) [1, 24]. These fragility analyses, concurrently with a *p*‐value, add intuitive insight into the robustness of a study's outcomes. A study's results are considered more fragile or less robust as the FI decreases. It is most effectively used in randomised control trials with a 1:1 allocation [26].

FI use has been increasing in popularity to add another layer of understanding to the results of previously published data. Reviews of published RCTs have uncovered a wide range of statistical fragility in orthopaedic research [14, 15, 18, 19, 20, 23, 29, 32, 41, 42, 53], with an average FI for orthopaedic literature of 3.5 [49]. Reviews of specific topics in knee and hip arthroplasty have uncovered average FIs ranging between 1 and 4 [15, 18, 20, 23, 42, 53]. The purpose of this study is to assess studies evaluating patellar resurfacing in TKA and determine the robustness of existing evidence using the FI.

## METHODS

A literature search of three databases, PubMed, MEDLINE and EMBASE, was performed according to the Preferred Reporting Items for Systematic Reviews and Meta‐Analyses (PRISMA) guidelines. The search strategy terms used were ‘knee arthroplasty’ [Mesh] AND ‘patellar resurfacing’ AND ‘randomised control trial’ OR ‘randomised controlled trial’ OR ‘RCT’. To be eligible for inclusion, studies needed to be designed and conducted as RCTs comparing TKA‐patellar surfacing with non‐patellar surfacing, with dichotomous outcomes reported and associated *p* values. Studies were excluded if they were systematic reviews, meta‐analyses, case reports, studies with greater than two intervention groups and studies with no dichotomous outcomes. Based on these eligibility criteria, two reviewers (TP and ED) independently screened all articles selected for full‐text review. All discrepancies were resolved with a third author (AH). Data collected from selected studies included year of publication, journal, sample size, power analysis, mean follow‐up, loss to follow‐up, dichotomous outcomes and associated *p*‐value for each outcome. Non‐dichotomous continuous data were not included in the analysis.

Outcomes were divided based on *p‐*value significance, with *p‐*value significance set as less than or equal to 0.05 and were divided into the following categories: anterior knee pain, any revision, any knee pain, patella‐related revision, patient satisfaction, any complication and patella‐related complication. Fragility indices and reverse fragility indices (hereafter, collectively referred to as FIs and FQs) were calculated utilising the package ‘fragility’ in R studio [27]. Groups were kept consistent across analyses, with the resurfaced groups labelled as experimental and the unresurfaced groups labelled as the control groups. Mean, median and interquartile range (IQR) for FI and FQ were calculated for the entire data set, as well as by study, by outcome significance and by outcome category. In addition, for the outcome category of anterior knee pain, FI and FQ were calculated by outcome significance.

## RESULTS

Of the 78 studies selected for full‐text review, 21 studies were included after screening and application of the inclusion and exclusion criteria (Table 1). These RCTs included 3910 unique patients. Several RCTs included were follow‐up analyses of an initial patient cohort [9, 11, 33]. Overall, including all outcome categories, the mean FI was 5.0 with a median FI of 5.65 (IQR 4.0−6.0), ranging from 1 to 31. The mean FQ was 0.044 with a median FQ of 0.048 (IQR 0.022−0.065). Data for each of the 21 studies are summarised in Table 1. Loss to follow‐up and deaths exceeded the median FI in 95% (18/19) of the studies with available data. The loss of follow‐up alone exceeded the median FI in 63% of studies (12/19).

**Table 1:** Study data (mean, median, IQR).

Study	# of outcomes	FI	FQ	Length of follow‐up	# of subjects/knees	Deaths and loss to follow‐up
Aunon 2016 [2]	1	7	0.054	3 years	115/129	3 lost to follow‐up
Barrack 1997 [5]	2	3.0	0.025	Mean 10 years	86/118	Not reported
Barrack 2001 [4]	3	4.3, 6.0, 3.5−6.0	0.047, 0.065, 0.038−0.065	Minimum 5 years	86/118	13 deaths, five excluded from follow‐up
Beaupre 2012 [6]	2	4.0, 4.0, 3.5−4.5	0.105, 0.105, 0.092−0.118	10 years	38/38	6 deaths, nine lost to follow‐up
Breeman 2011 [7]	2	2.0, 2.0, 1.5−2.5	0.001, 0.001, 0.001−0.001	5 years	1715/1715	181 deaths, 184 lost to follow‐up, 40 withdrawn
Burnett 2004 [11]	4	4.3, 4.0, 3.8−4.5	0.063, 0.058, 0.045−0.076	Mean 10.8 years	90/100	36 deaths, two unable to attend clinic visit
Burnett 2007 [9]	4	5.5, 5.5, 5.0−6.0	0.098, 0.098, 0.089−0.107	Mean 10 years	32/64	8 deaths, four withdrawn
Burnett 2009 [10]	5	5.0, 5.0, 4.0−5.0	0.058, 0.051, 0.042−0.064	Minimum 10 years	86/118	24 deaths, 12 lost to follow‐up, four excluded
Campbell 2006 [12]	9	4.4, 5.0, 4.0−5.0	0.065, 0.060, 0.049−0.086	10 years	100/100	22 deaths, 20 excluded or lost to follow‐up
Deroche 2022 [16]	5	5.4, 5.0, 4.0−6.0	0.024, 0.022, 0.017−0.026	Mean 17.6 months	245/250	4 excluded, 17 lost to follow‐up
Dong 2018 [17]	3	6.7, 7.0, 6.5−7.0	0.069, 0.073, 0.068−0.073	Mean 33.5 months	53/106	5 lost to follow‐up (10 knees)
Ha 2019 [22]	2	5.5, 5.5, 4.3−6.8	0.046, 0.046, 0.035−0.056	Minimum 5 years	66/132	1 death, five lost to follow‐up
Liu 2012 [28]	2	6.5, 6.5, 6.3−6.8	0.049, 0.049, 0.047−0.051	Minimum 7 years	133/133	None reported
Mayman 2003 [31]	1	4	0.040	10 years	90/100	29 deaths
Murray 2014 [33]	2	16.5, 16.5, 12.8−20.3	0.010, 0.010, 0.008−0.012	Median 10 years	1715/1715	457 deaths; 299 lost to follow‐up
Roberts 2015 [39]	11	5.9, 6.0, 4.5−7.0	0.033, 0.034, 0.020−0.047	10 years	255/327	62 deaths, one lost to follow‐up, nine withdrawn
Smith 2006 [43]	1	6	0.146	12−18 months	34/41	9 unsuitable for follow‐up
Smith 2008 [44]	3	4.7, 4.0, 3.5−5.5	0.028, 0.025, 0.022−0.032	Minimum 3 years	142/181	10 deaths, seven withdrawn, two lost to follow‐up
Thiengwitt‐ayapom 2019 [45]	2	3.0, 3.0, 2.0−4.0	0.038, 0.038, 0.025−0.050	1 year	84/84	4 lost to follow‐up
Waters 2003 [51]	3	13.0, 7.0, 4.0−19.0	0.028, 0.015, 0.009−0.040	Mean 5.3 years	431/514	42 deaths, seven lost to follow‐up
Wood 2002 [52]	4	5.3, 5.0, 3.8−6.5	0.024, 0.023, 0.017−0.030	Minimum 1 year	201/220	11 deaths and loss to follow‐up, nine were excluded from the analysis

Abbreviations: FI, fragility index; FQ, fragility quotient; IQR, interquartile range.

Stratified by the significance of outcomes, the median FI and FQ of nonsignificant findings are 3.0 (IQR 3.0−8.0) and 0.025 (IQR 0.014−0.065), respectively. In other words, at least half of the nonsignificant results only need three outcome reversals or 2.5% to become statistically significant (Table 2). For statistically significant findings, the median FI and FQ are 5.0 (IQR 4.0−6.0) and 0.045 (IQR 0.0022−0.064), respectively, indicating that a reversal of five events is needed for an event to change from being statistically significant to nonsignificant.

**Table 2:** Significant versus nonsignificant outcomes (mean, median, IQR).

	# of outcomes	FI	FQ
Significant	5	5.4, 5.0, 4.0−6.0	0.049, 0.045, 0.022−0.064
Nonsignificant	66	9.2, 3.0, 3.0−8.0	0.035, 0.025, 0.014−0.065

Abbreviations: FI, fragility index; FQ, fragility quotient; IQR, interquartile range.

The median FI of the seven individual outcome categories ranges from 4.0 to 7.0, while the median FQ ranges from 0.017 to 0.057 (Table 3). The primary outcome in most studies is the presence of anterior knee pain, in which 85% (17/20) of the studies found no statistically significant differences. Table 4 shows the fragility analysis for anterior knee pain, demonstrating a median FI of 6.0 (IQR 4.0−6.0) and median FQ of 0.060 (IQR 0.025−0.065). In the remaining three studies, which reported a lower prevalence of anterior knee pain in patients receiving patellar resurfacing with TKA, the median FI is 3.0 (IQR 3.0−17.0), and median FQ is 0.025 (IQR 0.019−0.045).

**Table 3:** FI by category (mean, median, IQR).

	# of outcomes	FI	FQ
Anterior knee pain	20	6.4, 6.0, 4−6	0.057, 0.057, 0.025−0.065
Any revision	10	5.4, 5.0, 4.0−7.0	0.048, 0.039, 0.035−0.065
Any knee pain	9	6.2, 5.0, 5.0−9.0	0.055, 0.044, 0.034−0.060
Patella‐related revision	10	4.2, 4.5, 2.3−5.8	0.032, 0.017, 0.007−0.057
Satisfaction	7	4.1, 4.0, 3.0−5.5	0.040, 0.049, 0.022−0.059
Any complication	5	9.6, 7.0, 5.0−7.0	0.059, 0.054, 0.022−0.073
Patella‐related complication	10	4.4, 4.5, 2.5−6.0	0.040, 0.037, 0.017−0.050

Abbreviations: FI, fragility index; FQ, fragility quotient; IQR, interquartile range.

**Table 4:** FI for anterior knee pain by outcome significance (mean, median, IQR).

	# of outcomes	FI	FQ
Nonsignificant	17	5.4, 6.0, 4.0−6.0	0.061, 0.060, 0.025−0.065
Significant	3	12.3, 3.0, 3.0−17.0	0.035, 0.025, 0.019−0.045

Abbreviations: FI, fragility index; FQ, fragility quotient; IQR, interquartile range.

## DISCUSSION

To our knowledge, this systematic review and fragility analysis is the first to examine the statistical robustness of RCTs comparing the dichotomous outcomes of patellar resurfacing compared to non‐patellar resurfacing in TKA. Available published RCTs on patellar resurfacing have an overall median FI of 5, with an FQ of 0.048. In other words, a reversal of only five outcome events or 4.8% of events within these studies, would be enough to change the significance of the findings. Specifically, the primary outcome of anterior knee pain has a median FI of 6.0 (IQR 4.0−6.0) and a median FQ of 0.057 (IQR 0.025−0.065). Presently, there are no defined thresholds for FI or FQ values that differentiate between statistically robust and fragile studies. However, analysing values with the number of patients lost to follow‐up can offer insights into the declared significance of statistical outcomes. For RCTs on patellar resurfacing, the loss to follow‐up exceeded the overall FI in 63% of studies.

The decision to perform patellar resurfacing during primary TKA remains controversial, with advantages and disadvantages for each approach. A systematic review, examining 33 RCTs involving 5499 primary TKAs found that patellar resurfacing leads to reduced risk of anterior knee pain, revision surgery and complications; however, there remains no difference in patient‐reported outcome measures between routine resurfacing, routine non‐resurfacing and selective resurfacing [21]. Many surgeons elect to perform selective patellar resurfacing based on factors such as the presence or location of patellar articular cartilage wear, the presence of inflammatory arthritis, native patella thickness and the level of preoperative anterior knee pain [34]. However, data on selective resurfacing remain mixed with lower and higher revision rates compared to always patellar resurfacing [30, 40, 48]. A study covering over 800,000 primary TKAs highlighted the significantly increased risk of all‐cause revision at 10 years with no resurfacing compared with resurfacing, leading to 2842 revisions compared with all TKAs having undergone resurfacing initially [46]. A cost‐effectiveness analysis of patellar resurfacing was also equivocal. One study found that always resurfacing resulted in more quality‐adjusted life years (QALYs) and was cheaper overall, as the incremental costs of resurfacing during the initial TKA were outweighed by the costs of complications and revisions needed to resurface some of the non‐resurfaced group [21]. However, Zmistowski et al. reported that patella resurfacing slightly improved QALYs in TKA at an incremental cost of $3032 per QALY, which escalated to $183,584 per QALY in cases with no patellar arthritis, indicating varied cost‐effectiveness based on patient selection and arthritis patterns [54].

The fragility of historical trial data can help us understand the FI and FQ values reported in our study. Although there are no objective cutoffs for adequate values, the median FI of 5 for patellar resurfacing TKA RCTs indicates that the literature is more fragile when compared to a median FI of 8 reported from an analysis of 399 general topic trials from high‐impact journals [50]. Looking specifically at fragility analyses for arthroplasty, a systematic review of RCT data for total joint arthroplasty reported an overall FI of 4 and 7 for significant and nonsignificant outcomes, respectively [23]. A fragility analysis on outcomes deemed as ‘strong evidence’ and statistically significant according to the clinical practice guidelines of the American Academy of Orthopaedic Surgeons identified a median FI of 2 and FQ of 0.022 [13]. Zabat et al. recently published an overall median FI of 3.0 for significance and nonsignificant outcomes from seven RCTs comparing robotic‐assisted and conventional TKAs [53]. Despite significant *p*‐values, a number of RCTs in orthopaedic journals have statistically fragile results. Future studies should strive to increase study power and report on individual fragility indices.

Interpreting FI values should include consideration of the nature of the intervention and the severity of potential outcomes. Previous research suggests that the FI threshold should balance the need for robust results against the risk of exposing patients to inferior treatments [3]. A lower FI may be deemed acceptable for interventions where the stakes involve survival or severe complications, as increasing the study's power could expose patients to unnecessary risk. Conversely, as in elective arthroplasty, a higher FI threshold might be used to establish statistical significance. Our study calculated a median FI of 7.0 and 5.0 for RCTs results comparing the rate of any complication and any revision, respectively, after patellar resurfacing. A higher FI and FQ highlight that complication reporting was notably more robust than satisfaction and revision reporting, with an FQ for complication outcomes at 0.054, higher than the FQs of 0.049 and 0.039 for satisfaction and revision rates, respectively. Comparison of patella‐related revisions was less robust, with an FQ of 0.017, meaning only 1.7% of event reversals in the study would change the statistical significance. These differences in fragility values suggest that the clinical significance of a reported *p*‐value can vary significantly within a single study, depending on the nature of the outcomes and underlines the importance of incorporating fragility assessments alongside *p*‐values in research. Our analysis found a greater number of nonsignificant outcomes (66) than significant outcomes (5); however, the significant outcomes had a greater median FQ, indicating greater statistical stability. Out of the significant outcomes, the three concerning anterior knee pain all favoured resurfacing. Taken together, these findings indicate that primary patellar resurfacing may be beneficial if persistent anterior knee pain is of clinical concern to the patient and surgeon, but patellar resurfacing may not affect additional outcomes, and the choice to resurface can be left to surgeon preference. However, this conclusion is limited by the statistical fragility of the analysed outcomes and not reflective of the extent of preoperative patellar arthritis; thus, larger RCTs for this comparison are necessary to decisively compare outcomes between resurfaced and unresurfaced patients, particularly in cases where there is little or no patellar arthritis.

Limitations of the study include that our literature search generated only 21 RCTs on patellar resurfacing that met inclusion criteria, and the RCTs presented a limited number of dichotomous outcomes for analysis. The necessity of dichotomous outcomes also limits the ability to perform fragility analysis on outcomes such as postoperative range of motion, patient‐reported functional outcomes and visual analogue pain scores and implant survivorship, which are usually reported as continuous quantitative variables. Another limitation is that a third of the studies did not describe a power analysis, so there is a risk that the primary outcomes in those studies and our fragility analysis may not be adequately powered. To reduce this limitation, the analysis included only outcomes specified in the research questions and for which statistical significance was determined. Although the included studies were all RCTs with low risk of selection and allocation bias, a formal assessment of bias using the revised Cochrane risk of bias tool was not performed. Lastly, there are no defined FI and FQ thresholds for interpreting the significance of the values, and further discourse is necessary for the application of fragility analysis in clinical decision‐making.

## CONCLUSION

The RCTs comparing patellar resurfacing to non‐patellar resurfacing in TKA demonstrate statistical fragility, with a few outcome event changes able to alter the statistical and clinical findings of these studies. We recommend the inclusion of FI and FQ in reporting alongside the *p* value and statistical significance of RCT results to improve the interpretability and clinical relevance in the TKA literature.

## AUTHOR CONTRIBUTIONS

Jess H. Lonner, Teja Polisetty and Alexandra L. Hohmann contributed to the study's conception and design. Material preparation and data collection were performed by Teja Polisetty and Eric DiDomenico. Analysis was performed by Alexandra L. Hohmann. Manuscript preparation was performed by Teja Polisetty, Eric DiDomenico and Alexandra L. Hohmann. Manuscript editing was performed by Jess H. Lonner. All authors read and approved the final manuscript.

## CONFLICTS OF INTEREST STATEMENT

Jess H. Lonner has the following conflicts of interest to disclose: American Association of Hip and Knee Surgeons: Board or committee member; Force Therapeutics: Paid consultant; Research support; Stock or Stock Options; Knee Society: Board or committee member; Pacira Pharmaceuticals: Paid consultant; Proteonova: Stock or Stock Options; Saunders/Mosby‐Elsevier: Publishing royalties, financial or material support; Smith & Nephew: IP royalties; Paid consultant; Research support; Springer: Publishing royalties, financial or material support; Wolters Kluwer Health—Lippincott Williams & Wilkins: Publishing royalties, financial or material support; Zimmer Biomet: IP royalties; Paid consultant; Paid presenter or speaker; Research support. The remaining authors declare no conflict of interest.

## ETHICS STATEMENT

This study only included a review of the existing literature for statistical outcomes with no involvement of human subjects; therefore, approval or exemption with our institutional review board and informed consent of human subjects was not required.

## Data Availability

Data analysed in this study are available from the corresponding author (Jess H. Lonner, MD, Jess.Lonner@rothmanortho.com) with a reasonable request.
